# Long non-coding RNA CASC7 is a promising serum biomarker for hepatocellular carcinoma

**DOI:** 10.1186/s12876-023-02961-7

**Published:** 2023-09-21

**Authors:** Ling Liao, Xia Chen, Hengliu Huang, Yuwei Li, Qing Huang, Zhen Song, Jie Luo, Tao Yuan, Shaoli Deng

**Affiliations:** 1 Department of Laboratory Medicine, Daping Hospital, Army Medical University, Third Military Medical University, No. 10, Changjiang Zhilu, DaPing, Yuzhong District, Chongqing, 400042 China; 2 Department of Clinical Laboratory, Chongqing University Hospital, Chongqing University, Chongqing, 400044 China; 3 Department of Laboratory Medicine, The First Branch of the First Affiliated Hospital of Chongqing Medical University, Chongqing, China; 4 Department of Clinical Laboratory, The 954th Hospital of Chinese People’s Liberation Army, No. 80, Naidong Road, Naidong District, Shannan, 856000 China; 5 Department of Hepatobiliary Surgery, Daping Hospital, Army Medical University, Third Military Medical University, No. 10, Changjiang Zhilu, DaPing, Yuzhong District, Chongqing, 400042 China

**Keywords:** CASC7, Hepatocellular carcinoma, Diagnosis, Serum, Biomarker

## Abstract

**Background:**

At present, a large number of studies have found that long non-coding RNAs (lncRNAs) can be used as biomarkers for diagnosis and monitoring prognosis of hepatocellular carcinoma (HCC). The expression of lncRNA cancer susceptibility candidate 7 (CASC7) in HCC has rarely been studied. The purpose of this study was to explore the expression of CASC7 and its correlation with clinical features, and to further analyze its diagnostic value in HCC.

**Methods:**

Serum samples were collected from 80 patients with HCC, 80 patients with chronic hepatitis B (CHB), and 80 healthy people. The expression level of serum CASC7 was detected by droplet digital PCR. Appropriate parametric and nonparametric tests were used for data analysis.

**Results:**

The results showed that the expression of CASC7 in serum of patients with HCC was significantly higher than that of patients with CHB (median: 8.8 *versus* 2.2 copies/µl, *p* < 0.001) and healthy controls (median: 8.8 *versus* 3.8 copies/µl, *p* < 0.001). High expression of serum CASC7 was significantly correlated with tumor number (*p* = 0.005), intrahepatic metastasis (IM) (*p* < 0.001), tumor size (*p* = 0.007) and tumor-node-metastasis (TNM) stage (*p* = 0.008). The area under the curve (AUC) of CASC7 to distinguish HCC patients from CHB patients and healthy controls was 0.808 (95% CI: 0.742–0.874) at the cut-off value of 7.24 copies/µl with 63.8% sensitivity and 95.2% specificity.

**Conclusions:**

This study suggested that CASC7 was significantly up-regulated in serum of patients with HCC and closely related to tumor number, IM, tumor size and TNM stage, which may serve as a promising diagnostic biomarker.

**Supplementary Information:**

The online version contains supplementary material available at 10.1186/s12876-023-02961-7.

## Introduction

Hepatocellular carcinoma (HCC) is the fourth most common cause of cancer-related death worldwide. Most cases of HCC (approximately 80%) occur in low-resource and middle-resource countries, particularly in African and Asian countries where medical and social care resources are often limited [1–3]. The mortality rate of HCC is extremely high, mainly due to the fact that it is usually found in the middle to late stage, and the metastasis of cancer cells and the recurrence after surgery also lead to the high mortality rate [4–6]. In the past two decades, the most commonly used clinical marker of HCC was alpha fetoprotein (AFP). However, about 20% ~ 40% of HCC patients show negative AFP, which is easy to cause missed diagnosis [7]. In addition, elevated AFP concentrations were detected in cirrhosis, hepatitis, cholangiocarcinoma, and testicular germ cell tumor [8]. Therefore, finding effective biomarkers and therapeutic targets will be the key to improve the level of diagnosis and clinical treatment of HCC.

Long non-coding RNAs (lncRNAs) is a non-coding RNA with a length of more than 200 nucleotides. Due to the lack of functional open reading frames that do not encode proteins or only encode short peptides, these lncRNAs were considered as “noise” in the human genome sequence by researchers and were ignored for a long time. However, with the further research on the functionology of lncRNAs, more and more research evidences show that lncRNAs play an important role in the development of human diseases [9–11]. Cancer Susceptibility Candidate 7 (CASC7) is a 9.3 kb length of lncRNA located in chr8: 140,520,156 − 140,529,501. CASC7 is a kind of dual-localized lncRNA expressed in the form of nuclear components, and associated with cytoplasmic polysomal complexes, suggesting that CASC7 plays a role in translational regulation [12]. In recent years, CASC7 has been reported in neuroblastoma [13], glioma [14], colorectal cancer [15] and nonsmall cell lung cancer [16]. Furthermore, the latest research showed that CASC7 were increased in HCC cells and tissues, which promoted tumor growth as well as HCC cell proliferation, invasion and migration [17]. And more notably, CASC7 was up-regulated in plasma of patients with heart failure and showed promising efficiency as a biomarker for the diagnosis of heart failure [18]. However, there are few studies on the expression of CASC7 in the serum of HCC patients and whether CASC7 can be used as a diagnostic biomarker for HCC.

Here, the serum levels of CASC7 in HCC patients, chronic hepatitis B (CHB) patients and healthy controls groups were detected by droplet digital PCR (ddPCR), and the expression differences of CASC7 in these three groups were compared. Meanwhile, the relationship between CASC7 expression and clinicopathologic characteristics of patients with HCC was analyzed to estimate whether it was involved in malignancy development. Receiver operating characteristics curve (ROC curve) was used to analyze the diagnostic efficacy of serum CASC7 and AFP, and calculate their sensitivity and specificity.

## Materials and methods

### Patients and clinical specimens

This study was conducted in the Daping Hospital of the Army Medical University (Chongqing, China) and was approved by the Ethics Committee of Army Medical Center. All the patients included in this study provided written informed consent in advance. Peripheral blood samples from total 240 participants were recruited from April 2018 to December 2019. In detail, 80 patients with HCC, 80 patients with CHB and 80 healthy people matched in age and sex were included. All patients with HCC were confirmed by imaging and pathological biopsy. The HBV surface antigen of CHB patients was positive, ultrasound examination was negative, and the outpatient department was followed up for a long time. In healthy people, there was no abnormal examination of hepatitis, liver disease and biochemistry of liver function. Blood samples were obtained before surgery and cannot be collected after radiotherapy, chemotherapy or targeted therapy. For each patient, 5 ml of peripheral blood was obtained. Within 2 h, the serum sample was collected by centrifugation at 3500 rpm for 10 min. Then, the supernatants were transferred to 1.5 mL Eppendorf tubes and stored at − 80 °C for future RNA extraction.

### RNA isolation and cDNA synthesis

Total RNA was extracted from 250 µl serum using the trizol LS reagent (Invitrogen, USA) according to the manufacturer’s instructions. RNA concentration and purity were determined using a NanoDrop 1000 UV spectrophotometer (Thermo, Wilmington, DE, USA). Reverse transcription of cDNA can be performed only when each sample contained 3000 ng RNA and the optical density ratio (260/280) is between 1.8 and 2.0. The EvoScript Universal cDNA Master reverse transcription kit (Roche, Basel, Switzerland) was used to obtain cDNA. The reaction conditions for the synthesis of cDNA were as follows: 15 min at 42℃, 5 min at 85℃, 15 min at 65℃ and 5 min at 4℃. The obtained cDNA was stored at − 80 °C for future use.

### Droplet digital PCR (ddPCR)

The QX200 droplet digital PCR system (Bio-Rad, Hercules, CA, USA) was used for CASC7 detection according to the manufacturer’s protocol. 10 µl 2x ddPCR Supermix for Probes (Bio-Rad), 2 µl 2.5x target probe (Takara, Dalian, China), 1 µl 10x forward and reverse primers each (Sangon, Shanghai, China) and 6 µl cDNA template were configured into a 20 µl volumes ddPCR reaction system. The reaction volume of each 20 µl was then mixed with 70 µl droplet generation oil (Bio-Rad) and transferred into QX200 Droplet Generator (Bio-Rad) to generate a maximum of 20,000 droplets from each sample. The droplets generated from each sample were transferred to a 96-well plate and amplified on the PCR instrument. Cycling was at 95℃ for 10 min, followed by 40 cycles of 94℃ for 30 s and 60℃ for 1 min, and a final step at 98℃ for 10 min. A no-template control (NTC) or a blank control was included in every assay. The QX200 Droplet reader (Bio-Rad) was used to read the results. The sequences of the PCR primers for CASC7 were as follows: 5’ ACTCGTTGCAGTCTTGTGAACA 3’ (forward) and 5’ TGGAACTCAAAGACCATGCTT 3’ (reverse). The sequence of the PCR probe was FAM-5’ TGTGGTCTGTTTGATTCCTCGTCGCT 3’-BHQ1.

### Laboratory measurements and clinical features were collected

Liver function indicators including total protein (TP), albumin (ALB), total bilirubin (TBIL), aspartate transaminase (AST), alanine transaminase (ALT), alkaline phosphatase (ALP), gamma-glutamine transferase (γ-GT), lactate dehydrogenase (LDH), 5’-nucleotidase (5’-NT), α-L-fucosidase (AFU), and cholinesterase (CHE) were detected using an AU5800 automatic biochemical analyzer (Beckman Co., Ltd., USA). Serum AFP and carcino-embryonic antigen (CEA) values were determined using a luminex200 (Luminex Co., Texas, USA). White blood cell (WBC), platelet (PLT), monocyte and lymphocyte were analyzed on the Sysmex XN-9000 analyzer (Sysmex Corporation, Kobe, Japan). HBV DNA was measured using diagnostic Kit for Quantification of HBV DNA (Sansure Biotech Inc., Hunan, China). Clinical information, including age, sex, tumor number, cirrhosis, intrahepatic metastasis (IM), tumor size, Barcelona Clinic Liver Cancer (BCLC) classification, tumor-node-metastasis (TNM) stage, and Child-Pugh were collected at hospital admission.

### Statistical analysis

All data were processed with Statistical Package for Social Sciences version 20.0 software (SPSS Inc., Chicago, IL, USA) and GraphPad Prism 8.0.2 (GraphPad Software, La Jolla, CA, USA). Student’s t test was operated to analyze normally distributed data, while non-normally distributed variables were tested by Mann-Whitney U Test between the two groups. One-way ANOVA was operated to analyze normally distributed data, while non-normally distributed variables were tested by Kruskal Wallis Test between the three groups. Comparison of percentages was done using the Pearson Chi-square test or Fisher’s Exact Test. Spearman correlation coefficient was used to evaluate the correlation between variables, the strength of the correlation is determined by the magnitude of the absolute value of the correlation coefficient r [19]. The diagnostic value of CASC7 was estimated by calculating the area under ROC curve (AUC). Optimal cut-off value was selected based on the Youden index, defined as the largest difference between the sensitivity and 1-specificity, taken over all points on the ROC curve. The odds ratio (OR) and 95% confidence interval (CI) were estimated by logistic regression analysis. Kaplan–Meier analysis and the log-rank test were performed for overall survival (OS). *p* < 0.05 was considered to be statistically significant.

## Results

### CASC7 was up-regulated in the serum of patients with HCC

Characteristics of the studied participants are shown in Supplementary Table 1. A total of 240 participants were enrolled in the current study, including 80 HCC patients (70 males and 10 females) with a median age of 56 years (range 27 to 78 years old), 80 patients with CHB (61 males and 19 females) with a median age of 46 years (range 22 to 72 years old), and 80 healthy controls (70 males and 10 females) with a median age of 48 years (range 30 to 69 years old). Age and sex characteristics were no significant different among the three groups (HCC group, CHB group and normal group). The levels of AFP, CEA, TBIL, AST, ALT, ALP, γ-GT, LDH, 5’-NT, AFU, WBC and monocyte in HCC patients were significantly higher than those in patients with CHB and/or healthy group (*p* < 0.001). Conversely, the indexes of TP, ALB, CHE, PLT and lymphocyte in HCC patients were significantly lower than those in patients with CHB and/or healthy group (*p* < 0.001).

**Fig. 1:**
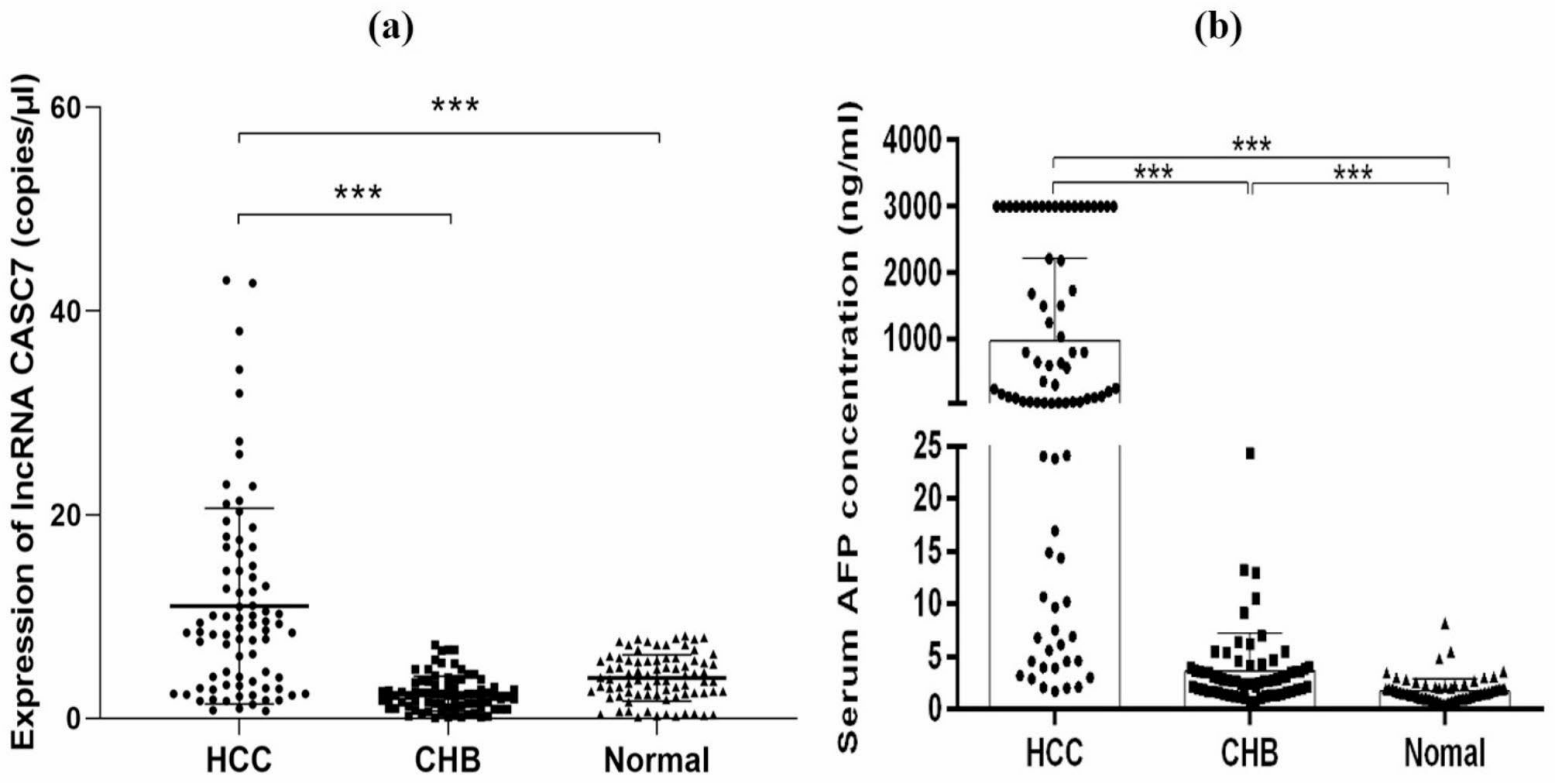
The expression levels of CASC7 and AFP in the serum of HCC, CHB and normal group. **(a)** Result showed that expression level of CASC7 was significantly up-regulated in HCC patients compared with CHB patients and healthy controls (*p* < 0.001***) and there was no significant difference between CHB patients and healthy controls. **(b)** Serum AFP concentration was significantly higher in HCC patients than CHB patients and healthy controls, and it was also higher in CHB patients than healthy controls (*p* < 0.001***). CASC7: cancer susceptibility candidate 7, AFP: alpha fetoprotein, HCC: hepatocellular carcinoma, CHB: chronic hepatitis B

The CASC7 expression levels in 80 HCC patients, 80 patients with CHB, and 80 healthy controls were detected by ddPCR, respectively. The results showed that, CASC7 level in HCC serum samples was much higher when compared to the patients with CHB (median: 8.8 *versus* 2.2 copies/µl, *p* < 0.001) and to the healthy controls (median: 8.8 *versus* 3.8 copies/µl, *p* < 0.001), but there was no significant difference between CHB group and normal group (*p* = 0.094) (Fig. 1a). Significantly higher AFP levels were detected in patients with HCC than patients with CHB (median: 155.2 *versus* 2.7 ng/ml, *p* < 0.001) and healthy controls (median: 155.2 *versus* 1.5 ng/ml, *p* < 0.001), and serum AFP concentration was also significantly higher in CHB patients than healthy controls (median: 2.7 versus 1.5 ng/ml, *p* < 0.001) (Fig. 1b).

### Serum CASC7 expression correlated with disease severity in HCC

**Fig. 2:**
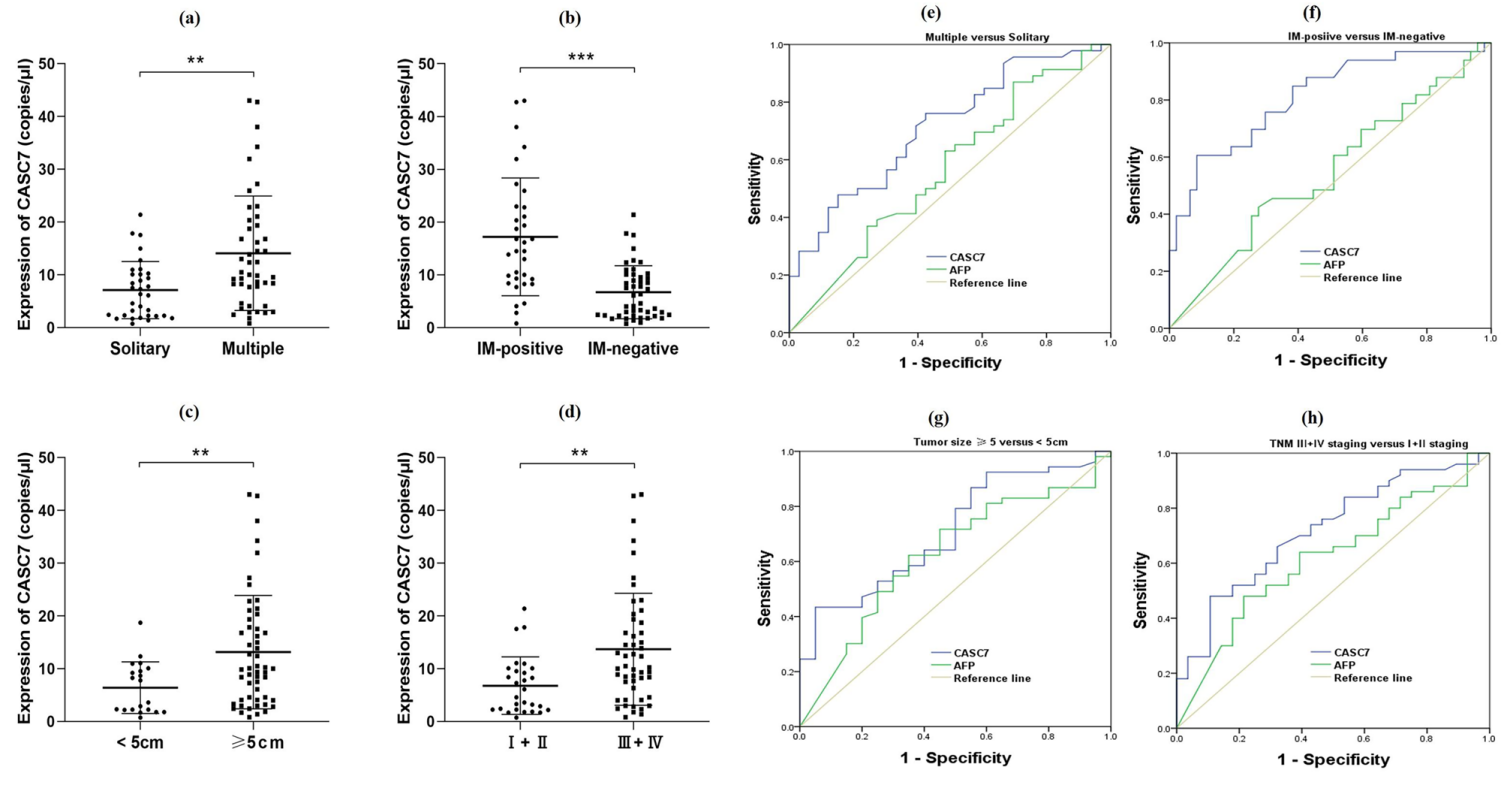
Relationship between the expression of CASC7 and tumor number, IM, tumor size, and TNM stage, and its diagnostic value. **(a)** Relationship between CASC7 expression and tumor number. **(b)** Relationship between CASC7 expression and IM. **(c)** Relationship between CASC7 expression and tumor size. **(d)** Relationship between CASC7 expression and TNM stage. **(e)** ROC curves of CASC7 and AFP for differential diagnosis of multiple tumors from solitary tumors. **(f)** ROC curves of CASC7 and AFP for differential diagnosis of IM-positive from IM-negative. **(g)** ROC curves of CASC7 and AFP for differential diagnosis of tumor size ≥ 5 from < 5. **(h)** ROC curves of CASC7 and AFP for differential diagnosis of TNM III + IV from TNM I + II. *p < 0.05, **p < 0.01, ***p < 0.001. CASC7: cancer susceptibility candidate 7, IM: intrahepatic metastasis, TNM: tumor node metastasis, ROC: receiver operating characteristic, AFP: alpha fetoprotein

Based on the significant up-regulation of CASC7 in HCC, we further analyzed the relationship between CASC7 and clinicopathological characteristics (Table 1). The result demonstrated that CASC7 expression was obviously influenced by tumor number (*p* = 0.005), IM (*p* < 0.001), tumor size (*p* = 0.007) and TNM stage (*p* = 0.008). We found that CASC7 was most closely associated with IM. A trend of higher CASC7 expression was also found in patients who had cirrhosis, higher BCLC classification and Child-Pugh grades, or were positive for HBV infection. The higher the serum CASC7 concentration, the more serious the disease. In addition, ROC curve was used to analyze the relationship between CASC7 and tumor number, IM, tumor size and TNM stage (Fig. 2; Table 2). To distinguish patients with multiple and solitary tumors, AUC was found to be 0.713 (95% CI: 0.600 to 0.826) for CASC7. ROC curve of CASC7 showed a strong separation between IM-positive and IM-negative with an AUC of 0.811 (95% CI: 0.714 to 0.909). In addition, serum CASC7 showed an AUC of 0.709 (95% CI: 0.583 to 0.836) to discriminate between tumor size ≥ 5 and < 5, and an AUC of 0.719 (95% CI: 0.604 to 0.834) to discriminate between patients with TNM III + IV from those with TNM I + II. Whereas, AFP could not discriminate between the clinicopathological parameters described above (*p* > 0.05).

**Table 1:** Correlation between CASC7 expression and clinicopathological characteristics of hepatocellular carcinoma

Clinical characteristics	Case number	lncRNA CASC7 expression	*p* value
Gender			0.844
Male	70	11.33 ± 10.31	
Female	10	10.56 ± 12.1	
Age (years)			0.132
40–60	45	11.03 ± 10.04	
< 40 or > 60	35	11.49 ± 11.14	
HBV infection			0.679
No	17	8.88 ± 8.72	
Yes	63	11.87 ± 10.87	
HBV-DNA (IU/ml)			0.152
< 100	17	9.32 ± 6.5	
≥ 100	63	11.75 ± 11.29	
AFP (ng/ml)			0.693
< 200	41	10.55 ± 11.2	
≥ 200	39	11.95 ± 9.74	
Tumor number			0.005^**^
Solitary	32	7.11 ± 5.51	
Multiple	48	13.99 ± 12.04	
Cirrhosis			0.559
No	35	10.77 ± 10.22	
Yes	45	11.22 ± 9.21	
Intrahepatic metastasis			< 0.001^***^
No	47	6.69 ± 5.03	
Yes	33	17.19 ± 11.16	
Tumor size			0.007^**^
< 5	20	6.39 ± 4.9	
≥ 5	53	13.13 ± 10.74	
BCLC			0.213
A + B	27	8.61 ± 8.18	
C + D	52	12.28 ± 10.2	
TNM stage			0.008^**^
I+II	28	6.77 ± 5.45	
III+IV	50	13.67 ± 10.61	
Child-Pugh			0.834
A	43	10.42 ± 9.1	
B	35	11.7 ± 10.5	
C	2	12.2 ± 5.6	

Note: Values are mean ± SD. **p < 0.01, ***p < 0.001

CASC7: cancer susceptibility candidate 7, HBV: hepatitis B virus, AFP: alpha fetoprotein, BCLC: Barcelona Clinic Liver Cancer, TNM: tumor node metastasis

**Table 2:** Comparisons of ROC curve parameters between CASC7 and AFP

Factors	Cut-off value	AUC (95%CI)	Sensitivity	Specificity	Youden index	*p* value
**Multiple** ***versus*** **Solitary**						
CASC7	7.6	0.713 (0.600-0.826)	76.10%	57.60%	0.337	0.001
AFP	8.6	0.567 (0.437–0.697)	87%	30.30%	0.173	0.311
**IM-positive** ***versus*** **negative**						
CASC7	12.84	0.811 (0.714–0.909)	60.60%	91.50%	0.521	< 0.001
AFP	915	0.545 (0.416–0.675)	42.40%	72.30%	0.147	0.491
**Tumor size ≥ 5*versus <*** **5**						
CASC7	12.36	0.709 (0.583–0.836)	43.40%	95%	0.384	0.006
AFP	107	0.623 (0.480–0.766)	62.30%	65%	0.273	0.107
**TNM III+IV** ***versus*** **I+II**						
CASC7	11.68	0.719 (0.604–0.834)	48%	89.30%	0.373	0.001
AFP	724	0.616 (0.488–0.744)	48%	78.60%	0.266	0.09
**HCC** ***versus*** **CHB**						
CASC7	7.24	0.853 (0.794–0.913)	63.80%	100%	0.638	< 0.001
AFP	13	0.927 (0.886–0.969)	75%	98.50%	0.735	< 0.001
**HCC** ***versus*** **Normal**						
CASC7	8.16	0.757 (0.678–0.836)	57.50%	100%	0.575	< 0.001
AFP	4	0.981 (0.966–0.997)	91.30%	96.30%	0.876	< 0.001
**HCC** ***versus*** **CHB and Normal**						
CASC7	7.24	0.808 (0.742–0.874)	63.80%	95.20%	0.590	< 0.001
AFP	5.57	0.957 (0.931–0.983)	85%	93.90%	0.789	< 0.001

ROC: Receiver operating characteristics, CASC7: cancer susceptibility candidate 7, AFP: alpha fetoprotein, AUC: area under the curve, CI: confidence interval, IM: intrahepatic metastasis, TNM: tumor node metastasis, CHB: chronic hepatitis B

**Fig. 3:**
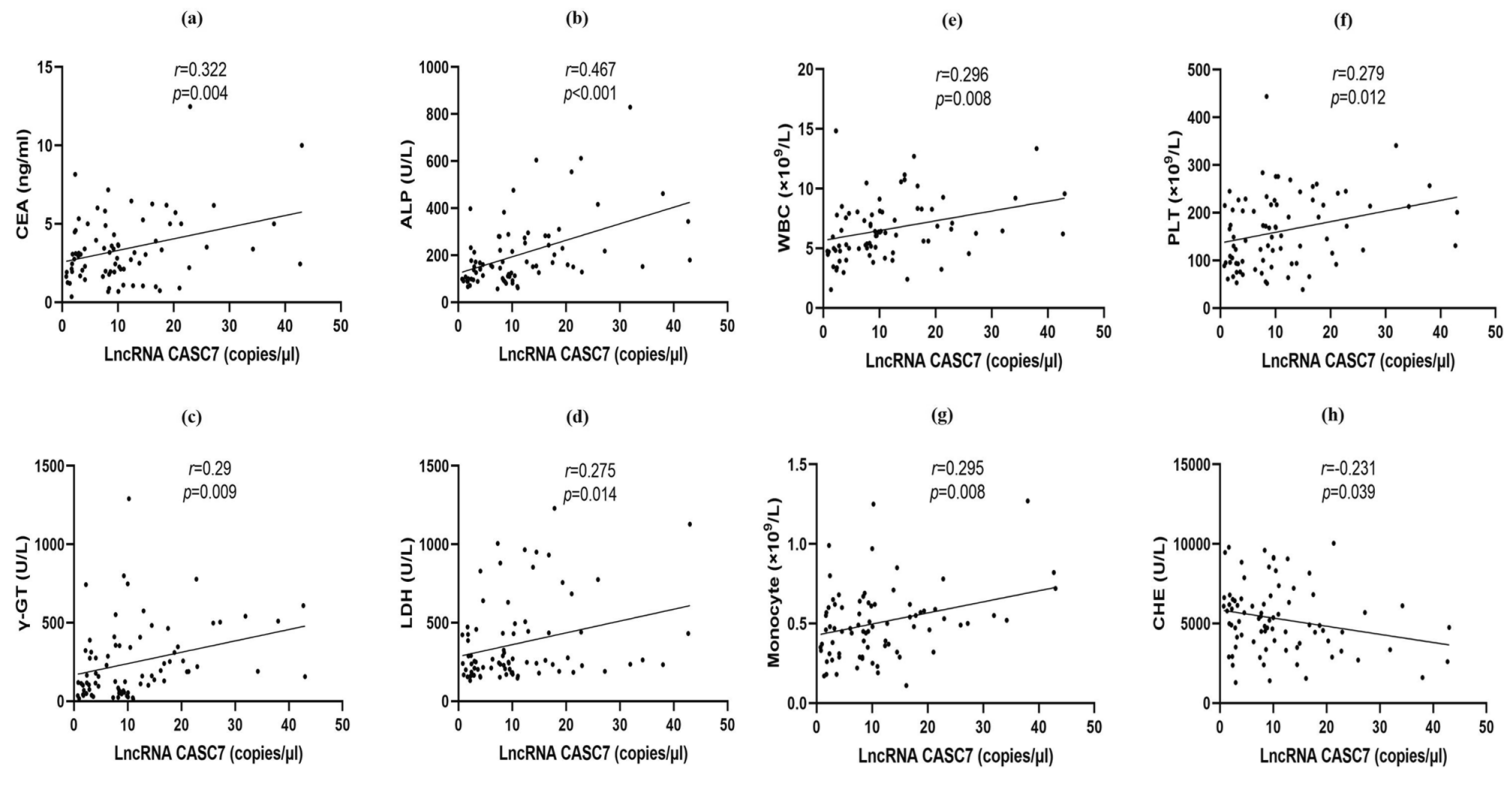
Correlation between serum CASC7 and biochemical features in 80 HCC patients. **(a-g)** Serum CASC7 concentration showed moderate positive correlations with CEA and ALP, and weak positive correlations with γ-GT, LDH, WBC, PLT and Monocyte. **(h)** Serum CASC7 concentration exhibited a weak negative correlation with CHE. *p* < 0.05 was considered to be statistically significant. CASC7: cancer susceptibility candidate 7, HCC: hepatocellular carcinoma, CEA: carcino-embryonic antigen, ALP: alkaline phosphatase, γ-GT: gamma-glutamine transferase, LDH: lactate dehydrogenase, WBC: white blood cell, PLT: platelet, CHE: cholinesterase

Correlation analysis of CASC7 with other clinical indicators showed moderate positive correlations with CEA (r = 0.322, *p* = 0.004) and ALP (r = 0.467, *p* < 0.001), and weak positive correlations with γ-GT (r = 0.29, *p* = 0.009), LDH (r = 0.275, *p* = 0.014), WBC (r = 0.296, *p* = 0.008), PLT (r = 0.279, *p* = 0.012) and Monocyte (r = 0.295, *p* = 0.008), and a weak negative correlation with CHE (r = − 0.231, *p* = 0.039) (Fig. 3). These results show that CASC7 expression was associated with known risk factors for HCC [20, 21].

### Diagnostic value of serum CASC7 and AFP for HCC

**Fig. 4:**
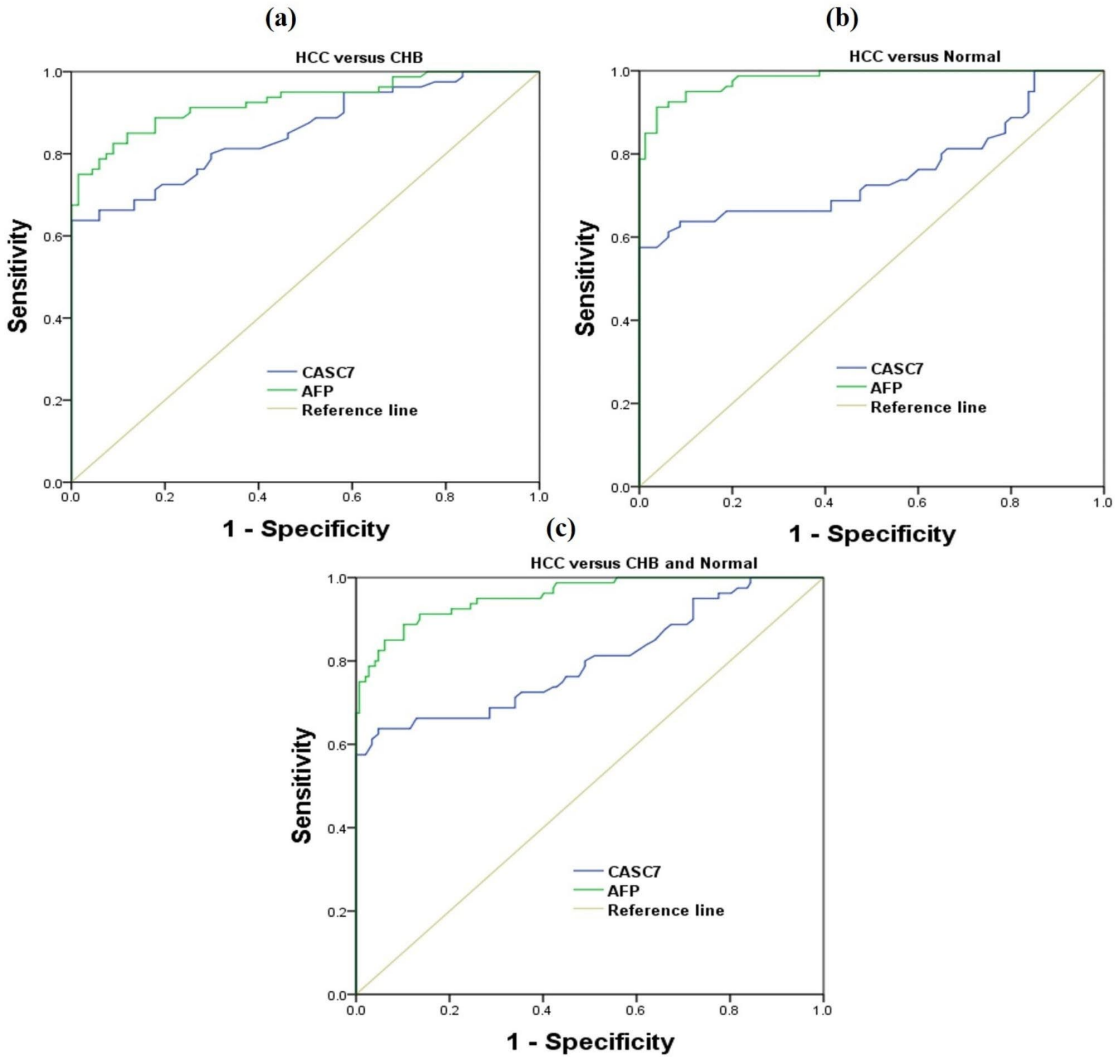
ROC curve analyses for identifying HCC patients from CHB patients and healthy controls. **(a)** ROC curve analyses of serum CASC7 and AFP for distinguishing HCC patients from CHB patients. **(b)** ROC curve analyses of serum CASC7 and AFP for distinguishing HCC patients from normal controls. **(c)** ROC curve analyses of serum CASC7 and AFP for distinguishing HCC patients from CHB patients and normal controls. ROC: receiver operating characteristic, HCC: hepatocellular carcinoma, CHB: chronic hepatitis B, CASC7: cancer susceptibility candidate 7, AFP: alpha fetoprotein

To explore the characteristics of CASC7 as a potential biomarker for HCC, we analyzed the ROC curve and the AUC of ROC for 80 HCC patients, 80 patients with CHB, and 80 healthy controls. Serum CASC7 showed an AUC of 0.853 (95% CI: 0.794–0.913) and 0.757 (95% CI: 0.678–0.836) to distinguish patients with cancer from CHB and normal individuals. As the most commonly used marker for evaluating HCC, serum AFP showed an AUC of 0.927 (95% CI: 0.886–0.969) and 0.981 (95% CI: 0.966–0.997) to distinguish patients with cancer from CHB and normal individuals. The diagnostic value of CASC7 and AFP in differentiating patients with HCC from non-cancer (CHB + normal) was further assessed. The AUC of CASC7 to distinguish HCC group from non-cancer group was 0.808 (95% CI: 0.742–0.874) at the cut-off value of 7.24 copies/µl with 63.8% sensitivity and 95.2% specificity. The AUC of AFP was 0.957 (95% CI: 0.931–0.983) with 85% sensitivity and 93.9% specificity, respectively, at a cut-off value of 5.57 ng/ml (Table 2; Fig. 4). It is worth noting that AFP has a better ability to distinguish between groups than CASC7.

Spearman rank correlation analysis of serum CASC7 and AFP levels in 80 HCC patients showed no correlation between CASC7 and AFP levels in HCC patients (r = 0.178, *p* = 0.114). We used logistic regression model to predict HCC, variable assignment was shown in Supplementary Table 2. The results indicated that CASC7 is a risk factor, and patients with high serum levels of CASC7 had a 1.358 times higher risk of developing HCC compared with patients with low serum levels (OR = 1.358, 95% CI: 1.159–1.591, p < 0.001). In addition, patients with high AFP level also had a 1.32-fold increased risk of HCC (OR = 1.320, 95% CI: 1.142–1.526, p < 0.001).

Furthermore, we explored the prognostic value of serum CASC7 in HCC using Kaplan–Meier survival curves. Based on the median of the overall expression level of lncRNA CASC7, we divided the 80 HCC patients into lncRNA CASC7 low expression group (47 cases) and high expression group (33 cases). Unfortunately, as 43 HCC cases could not be contacted, we only collected the survival of 37 HCC patients. Among them, 20 cases had low expression of lncRNA CASC7 and 17 cases had high expression. Although there was no significant difference in survival time between the two groups (HR = 2.139, 95% CI: 0.889–5.154, p = 0.069), there was a tendency for the survival time of the lncRNA CASC7 low expression group to be longer than that of the lncRNA CASC7 high expression group (Supplementary Fig. 1), which suggested that lncRNA CASC7 may have some value in determining the prognosis of HCC.

## Discussion

HCC is a common malignancy tumor worldwide, with a high recurrence and poor survival rate. However, the etiology and pathogenesis of HCC have not been fully clarified [22]. With the continuous progress of medical technology, comprehensive treatment of HCC has made some progress, but its therapeutic effect is still not optimistic, and the cumulative postoperative recurrence rate is as high as 70 − 80% [23, 24]. In the past few years, many studies have pointed out the potential use of lncRNAs as diagnostic markers or therapeutic targets for the treatment of HCC. For example, highly up-regulated in liver cancer (HULC) was identified as the first hepatocyte specific lncRNA, representing a prominent novel biomarker for HCC [25]. Yao et al. [26] found that lnc-FAM72D-3 and lnc-EPC1-4 may contribute to hepatocarcinogenesis and may be used as potential candidate biomarkers for the diagnosis of HCC. In another study, Yu et al. [27] found that the risk model based on three lncRNAs (RP11-150O12.3, RP11-187E13.1, and RP13-143G15.4) had a good survival prediction ability for HCC patients. Moreover, lncRNA PCA3 has been approved by FDA for clinical diagnosis of prostate cancer, so it is feasible to seek lncRNA in peripheral blood for diagnosis of HCC [28].

The development of nucleic acid quantitative methods is of great significance for disease diagnosis and disease process monitoring. With the development of science and technology, many methods of nucleic acid detection have been applied to the quantification of clinical samples, and real-time fluorescent quantitative PCR (qPCR) is the most widely used in the quantitative detection of clinical samples. In 1999, Bert Vogelstein and Kenneth W. Kinzler formally proposed the concept of digital PCR (dPCR) to quantitatively detect c-Ki-Ras mutations in fecal samples of colorectal cancer [29]. dPCR technology is a new nucleic acid quantification method, which converts the index signal of ordinary PCR into digital signal, so as to carry out the absolute quantification of the template. Compared with qPCR, dPCR does not require the establishment of a standard curve and is more accurate for the quantification of low concentration clinical samples. In our study, we used QX200 digital PCR instrument to detect the expression of serum lncRNA CASC7 in all samples under the same experimental conditions.

CASC7 is a noncoding RNA with a length of 9.3 kb, the function of which is largely unknown [12]. In fact, some cancer susceptibility candidate genes in the same family have been shown to be associated with the development of HCC. LncRNA CASC9 was expected to be a potential diagnostic and prognostic indicator for HCC [30]. Fan et al. [31] revealed that lncRNA CASC2 inhibited the viability and induced the apoptosis of HCC cells by regulating miR-24-3p. In this study, we investigated serum expression levels of lncRNA CASC7 in HCC patients compared to CHB patients and healthy individuals. Our results showed that a markedly higher serum CASC7 level was detected in patients with HCC than patients with CHB and healthy controls, and indicated that CASC7 may play a role in the pathogenesis of HCC.

To further explore whether it is involved in the development of HCC, we investigated the association of CASC7 with clinicopathological characteristics, and found that high expression of serum CASC7 was associated to varying degrees with multiple well known risk factors for HCC, including tumor number, IM, tumor size and TNM stage, suggesting its role in predicting progression and dynamics of tumor. Previous studies have shown that circulating lncRNAs have prognostic potential [32]. LncRNA-HEIH is associated with the recurrence of HBV-associated HCC, and high expression of lncRNA-HEIH predicts a worse prognosis [33]. Similarly, plasma HULC is significantly higher in patients with higher Edmondson histological grades [34]. Our results showed that high serum CASC7 level was associated with high degree of HCC malignancy and tend to lower survival time, suggesting that high serum CASC7 level may be an independent prognostic factor for poor prognosis of HCC patients. Metastasis is a key factor affecting the prognosis of patients with HCC and other tumors. The AUC of CASC7 to distinguish IM from non-IM was 0.811, indicating that CASC7 may be a favorable indicator for predicting IM, which provides a new idea for us to explore the molecular mechanism in the process of HCC metastasis.

To understand whether serum CASC7 can be used as a serological diagnostic indicator for HCC, we investigated the diagnostic potential of CASC7 by calculating the AUCs of serum CASC7 and AFP. It is worth noting that the AUC of AFP (0.957) is higher than the range level of previous studies [35–37]. Firstly, it might due to the deviation caused by the small number of samples included in this study. Secondly, HCC patients were mostly in the middle and advanced stages, which might also lead to the high AFP value. The ROC analysis indicated that serum CASC7 was of great value in distinguishing HCC patients from healthy controls, with AUC of 0.757 (57.5% sensitivity, 100% specificity). HBV prevalence is highly ecologically associated with HCC morbidity and mortality worldwide, which is a major risk factor for HCC in most of Asia and sub-Saharan Africa [38]. Recent studies have shown that some lncRNAs play an important role in HBV-related diseases, especially in the process of carcinogenesis, such as lncRNA Dreh, which inhibits the metastasis of hepatocellular carcinoma and is down-regulated by HBx, and lncRNA ZNRD1-AS1, which affects both HBV infection and the occurrence of hepatocellular carcinoma [39, 40]. In order to eliminate the possible influence of HBV infection on the expression of CASC7 in HCC group, we set CHB group as our disease control. The ROC results also showed that serum CASC7 was helpful for the differentiation of HCC patients and CHB patients, and its AUC was 0.853 (63.8% sensitivity, 100% specificity). The diagnostic value of CASC7 in distinguishing HCC patients from CHB patients and healthy control was demonstrated by ROC curve with an AUC of 0.808, a sensitivity of 63.8%, and a specificity of 95.2%. These data indicated that serum CASC7 might represent a potential diagnostic biomarker for HCC. Although AFP had better performance than CASC7, we found increased AFP in chronic active hepatitis in our study. Therefore, serum AFP still has some limitations as an important indicator of HCC. Further study of CASC7 in patients with early-stage HCC is critical to determine the diagnostic potential of CASC7 in HCC.

Although this study confirmed the value of CASC7 in HCC, there are still some limitations. Firstly, this study was limited by its small sample size, our results still need to be verified by large-scale multicenter studies. Secondly, the effect of CASC7 on survival in HCC patients may be biased due to the large amounts of censored data. Lastly, its mechanisms and other effects remain unknown. Hence, we hope to carry out basic research in the future to further analyze the way CASC7 affects the occurrence and development of HCC.

## Conclusions

In conclusion, the increased expression of CASC7 may be related to multiple clinical factors in the development of HCC. In clinical applications, peripheral blood detection has the advantages of repeatability and less trauma, so circulating CASC7 is expected to become an important marker for the diagnosis of HCC and the monitoring of disease progression.

### Electronic supplementary material

Below is the link to the electronic supplementary material.

Supplementary Material 1

## Data Availability

The datasets used and/or analysed during the current study are available from the corresponding author on reasonable request.

## Abbreviations

lncRNA: long non-coding RNA
HCC: hepatocellular carcinoma
CASC7: cancer susceptibility candidate 7
CHB: chronic hepatitis B
IM: intrahepatic metastasis
TNM: tumor-node-metastasis
AUC: area under the curve
AFP: alpha fetoprotein
ddPCR: droplet digital PCR
ROC curve: Receiver operating characteristics curve
NTC: no-template control
TP: total protein
ALB: albumin
TBIL: total bilirubin
AST: aspartate transaminase
ALT: alanine transaminase
ALP: alkaline phosphatase
γ-GT: gamma-glutamine transferase
LDH: lactate dehydrogenase
5'-NT: 5’-nucleotidase
AFU: α-L-fucosidase
CHE: cholinesterase
CEA: carcino-embryonic antigen
WBC: White blood cell
PLT: platelet
BCLC: Barcelona Clinic Liver Cancer
CI: confidence interval
HULC: highly up-regulated in liver cancer
qPCR: quantitative PCR
dPCR: digital PCR
