# Supplementary material for: Long non-coding RNA CASC7 is a promising serum biomarker for hepatocellular carcinoma
Source: BMC Gastroenterol. 2023 Sep 21;23:324. doi: 10.1186/s12876-023-02961-7 (PMC10514991; doi:10.1186/s12876-023-02961-7)
Supplement: Supplementary file 1 — Supplementary Material 1 [file 12876_2023_2961_MOESM1_ESM.docx]

**Supplementary Information**

**Supplementary Table 1** Characteristics of the HCC patients, CHB patients and healthy control groups

| **Characteristics** | **HCC group** | **CHB group** | **Normal group** | ***p* value** |
| --- | --- | --- | --- | --- |
|  | **n = 80** | **n = 80** | **n = 80** |  |
| Gender |  |  |  | 0.084^a^ |
| Male | 70 | 61 | 70 |  |
| Female | 10 | 19 | 10 |  |
| Age (years) |  |  |  | 0.260^~~a~~^ |
| 40 - 60 | 45 | 49 | 55 |  |
| < 40 or > 60 | 35 | 31 | 25 |  |
| AFP (ng/ml) | 155.2 (11.6, 2204.5) | 2.7 (1.8, 4) | 1.5 (1.1, 1.9) | < 0.001^b^ |
| CEA (ng/ml) | 3.1 (1.9, 4.5) | 1.7 (1.2, 2.5) | 1.5 (1.1, 2.3) | < 0.001^b^ |
| TP (g/L) | 69.5 (64.1, 74.5) | 74.4 (71.6, 78.1) | 73.5 (70.2, 75.3) | < 0.001^b^ |
| ALB (g/L) | 38.9 (32.9, 42.4) | 46.1 (43.5, 47.6) | 46.1 (45, 48.1) | < 0.001^b^ |
| TBIL (μmol/L) | 19.5 (15.0, 30.1) | 15.4 (12.0, 19.1) | 15.0 (12.6, 19.1) | < 0.001^b^ |
| AST (U/L) | 61.4 (40.2, 93.2) | 28.0 (22.6, 34.8) | 22.1 (19.4, 25.1) | < 0.001^b^ |
| ALT (U/L) | 43.8 (29.2, 71.7) | 29.5 (23.4, 38.3) | 19.7 (15.3, 27.9) | < 0.001^b^ |
| ALP (U/L) | 151.9 (99.4, 249.7) | 81.3 (64.2, 104.7) | - | < 0.001^c^ |
| γ-GT (U/L) | 161.5 (67.8, 352.7) | 19.3 (13.3, 31.9) | - | < 0.001^c^ |
| LDH (U/L) | 248.9 (190.7, 439.3) | 187.1 (145.1, 223.4) | - | < 0.001^c^ |
| 5'-NT (U/L) | 21.2 (11.7, 35.8) | 5.5 (4.8, 7.5) | - | < 0.001^c^ |
| AFU (U/L) | 42.1 (33.7, 59) | 27.1 (22, 30.3) | - | < 0.001^c^ |
| CHE (U/L) | 5065.5 (3777, 6499) | 8181 (7505, 9500) | - | < 0.001^d^ |
| WBC (×10^9^/L) | 6.2 (4.8, 8) | 5.2 (4.4, 6.6) | 5.5 (4.7, 6.5) | 0.081^b^ |
| PLT (×10^9^/L) | 150.5 (94.3, 216.8) | 170 (127, 222) | 197 (166, 250) | < 0.001^b^ |
| Lymphocyte (×10^9^/L) | 1 (0.7, 1.4) | 1.7 (1.2, 2.3) | 1.8 (1.5, 2.2) | < 0.001^e^ |
| Monocyte (×10^9^/L) | 0.5 (0.3, 0.6) | 0.4 (0.2, 0.4) | 0.3 (0.3, 0.4) | < 0.001^b^ |
| CASC7 (copies/μl) | 8.8 (3.2, 14.8) | 2.2 (1.1, 3.5) | 3.8 (2.3, 5.7) | < 0.001^b^ |

Note: Data are presented as number or median (25 percentiles, 75 percentiles). ^a^ Chi-square, ^b^ Kruskal Wallis Test, ^c^ Mann-Whitney U Test, ^d^ Independent-Samples T Test, ^e^ One-way ANOVA.

HCC: Hepatocellular carcinoma, CHB: chronic hepatitis B, AFP: [alpha](C:/Users/Administrator/AppData/Local/youdao/dict/Application/8.9.3.0/resultui/html/index.html#/javascript:;) [fetoprotein](C:/Users/Administrator/AppData/Local/youdao/dict/Application/8.9.3.0/resultui/html/index.html#/javascript:;), CEA: [carcino-embryonic](C:/Users/Administrator/AppData/Local/youdao/dict/Application/8.9.3.0/resultui/html/index.html#/javascript:;) [antigen](C:/Users/Administrator/AppData/Local/youdao/dict/Application/8.9.3.0/resultui/html/index.html#/javascript:;), TP: total protein, ALB: albumin, TBIL: total bilirubin, AST: aspartate transaminase, ALT: alanine transaminase, ALP: [alkaline](C:/Users/Administrator/AppData/Local/youdao/dict/Application/8.9.3.0/resultui/html/index.html#/javascript:;) [phosphatase](C:/Users/Administrator/AppData/Local/youdao/dict/Application/8.9.3.0/resultui/html/index.html#/javascript:;), γ-GT: gamma-glutamine transferase, LDH: lactate dehydrogenase, 5'-NT: 5'-nucleotidase, AFU: α-L-fucosidase, CHE: cholinesterase, WBC: white blood cell, PLT: platelet, CASC7: cancer susceptibility candidate 7.

**Supplementary Table 2** Logistic regression analysis to predict HCC

| **Factors** | **β** | ***p* value** | **OR** | **95%CI** |
| --- | --- | --- | --- | --- |
| LncRNA CASC7 | 0.306 | < 0.001 | 1.358 | 1.159 - 1.591 |
| AFP | 0.277 | < 0.001 | 1.320 | 1.142 - 1.526 |
| Constant | -4.512 | < 0.001 | 0.011 |  |

HCC: Hepatocellular carcinoma, CASC7: cancer susceptibility candidate 7, AFP: [alpha](C:/Users/Administrator/AppData/Local/youdao/dict/Application/8.9.3.0/resultui/html/index.html#/javascript:;) [fetoprotein](C:/Users/Administrator/AppData/Local/youdao/dict/Application/8.9.3.0/resultui/html/index.html#/javascript:;), OR: odds ratio, CI: confidence interval.


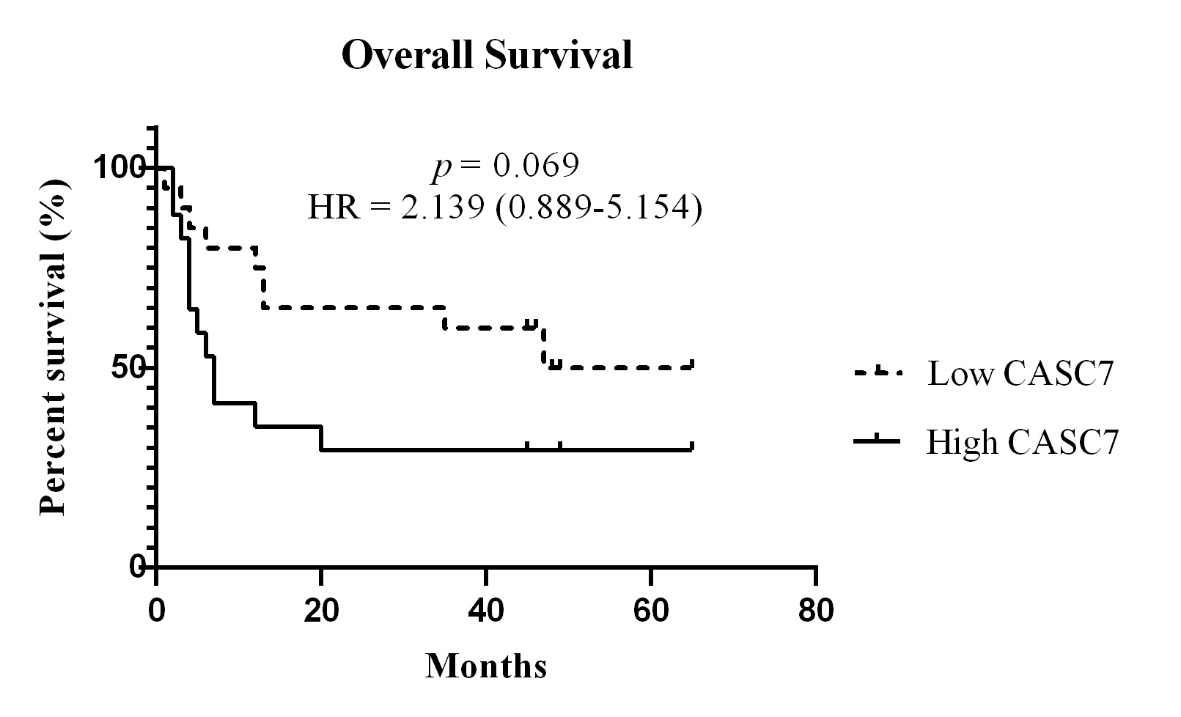


**Supplementary Fig. 1** LncRNA CASC7-based overall survival analysis of HCC. Based on the median of the overall expression level of lncRNA CASC7, the 80 HCC patients were divided into lncRNA CASC7 low expression group and high expression group. Log-rank test for statistical analysis. HR: hazard ratio.
